# Transforming Global Health by Improving the Science of Scale-Up

**DOI:** 10.1371/journal.pbio.1002360

**Published:** 2016-03-02

**Authors:** Margaret E. Kruk, Gavin Yamey, Sonia Y. Angell, Alix Beith, Daniel Cotlear, Frederico Guanais, Lisa Jacobs, Helen Saxenian, Cesar Victora, Eric Goosby

**Affiliations:** 1 Department of Global Health and Population, Harvard T.H. Chan School of Public Health, Boston, Massachusetts, United States of America; 2 Evidence to Policy Initiative, Global Health Group, University of California San Francisco, San Francisco, California, United States of America; 3 New York City Department of Health and Mental Hygiene, Long Island City, New York, United States of America; 4 Independent global health consultant, Arlington, Virginia, United States of America; 5 Health, Nutrition and Population, World Bank Group, Washington, District of Columbia, United States of America; 6 Social Protection and Health Division, Inter-American Development Bank, Lima, Peru; 7 Independent global health consultant, San Francisco, California, United States of America; 8 Results for Development, Washington, District of Columbia, United States of America; 9 International Center for Equity in Health, Federal University of Pelotas, Pelotas, Brazil; 10 Global Health Delivery and Diplomacy, Global Health Sciences, University of California San Francisco, San Francisco, California, United States of America

## Abstract

In its report *Global Health 2035*, the Commission on Investing in Health proposed that health investments can reduce mortality in nearly all low- and middle-income countries to very low levels, thereby averting 10 million deaths per year from 2035 onward. Many of these gains could be achieved through scale-up of existing technologies and health services. A key instrument to close this gap is policy and implementation research (PIR) that aims to produce generalizable evidence on what works to implement successful interventions at scale. Rigorously designed PIR promotes global learning and local accountability. Much greater national and global investments in PIR capacity will be required to enable the scaling of effective approaches and to prevent the recycling of failed ideas. Sample questions for the PIR research agenda include how to close the gap in the delivery of essential services to the poor, which population interventions for non-communicable diseases are most applicable in different contexts, and how to engage non-state actors in equitable provision of health services in the context of universal health coverage.

## Abstract 2

Kruk and colleagues examine the emerging field of policy and implementation research, the scientific enterprise aimed at closing "the delivery gap"—the gap between the interventions known to be effective and what is actually being delivered.

## Background: The Global Health Delivery Gap

**Fig 1:**
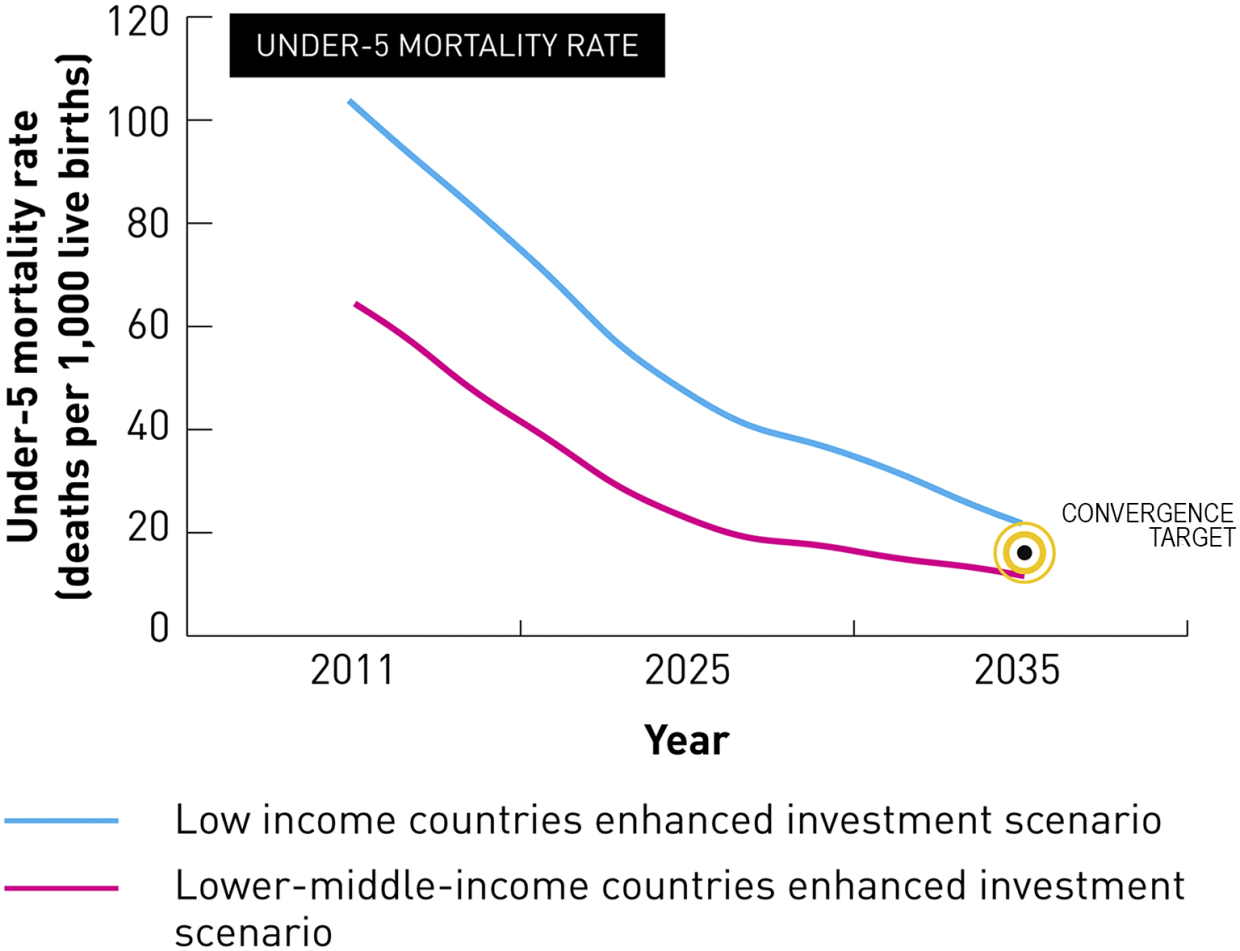
Impact of enhanced health investments on under-five mortality rate in low- and lower-middle-income countries. Data are from [1]. *Image credit*: *The Commission on Investing in Health*.

*Global Health 2035*, the report of the Commission on Investing in Health (CIH), laid out an ambitious roadmap for achieving dramatic improvements in health in low-income countries (LICs) and lower-middle-income countries (LMICs) over the next two decades through enhanced health sector investments [1]. Modeling by the CIH showed the technical and financial feasibility of achieving a “grand convergence” in global health—a reduction in infectious, child, and maternal mortality rates in almost all LICs and LMICs down to universally low levels (Fig 1). The public health impact of achieving a grand convergence would be impressive: 10 million deaths per year could be averted from 2035 onward.

Such an outcome would be technically possible through greater investments in scaling up:

- Existing medicines, vaccines, diagnostic tests, and other health tools to very high coverage levels (Table 1) [2];
- Public health and healthcare delivery systems for delivering these interventions, particularly in primary care; and
- The discovery and development of new health technologies, as discussed in the other five translational science essays in this collection.

**Table 1:** Examples of the scale-up of maternal and child health interventions required to achieve a grand convergence by 2035. Data from reference [2].

Intervention	Current coverage in low- and lower-middle-income countries	Coverage rate by 2035 that will be required to reach convergence
Modern family planning methods	30%	50%
Skilled birth assistance in labor	65%	99%
Neonatal resuscitation	28%	84%
Pregnant women sleeping under an insecticide-treated bed net for malaria prevention	26%	100%
Treatment of malaria in pregnant women	55%	100%
Kangaroo care (skin-to-skin contact for the newborn)	4%	95%
Oral rehydration therapy (ORT) for childhood diarrhea	40%	99%

Note: coverage is the percent of the population in need receiving the intervention.

Achieving convergence would cost around US$70 billion annually, over and above current health spending in LICs and LMICs (about US$25 billion per year in LICs and US$45 billion per year in LMICs) [1]. Currently, annual domestic spending on health is about US$26 billion by LICs and around US$217 billion by LMICs. Thus, the average annual incremental cost of achieving convergence would represent about a doubling of current spending in LICs and a 20% increase over current spending in LMICs. Most of the cost of convergence could be financed from the expected economic growth of these countries, which are on course to add about US$10 trillion to their GDP by 2035. The remainder could be financed through external aid. In Box 1, we show the CIH’s estimates of how convergence might be financed from now to 2035. Importantly, donors must be flexible in allowing health aid to be used to build national health systems platforms, provided that there is transparency in how these resources are being used.

**Box 1. Paying for the Grand Convergence: The Mix of Domestic Versus Donor Spending**

The CIH report provides illustrative estimates for how convergence could be financed by a mix of spending by national governments and external donor assistance. Currently, annual total donor assistance for health is about US$30 billion, and annual government spending on health is around US$26 billion by LICs and around US$217 billion by LMICs.

**Fig 2:**
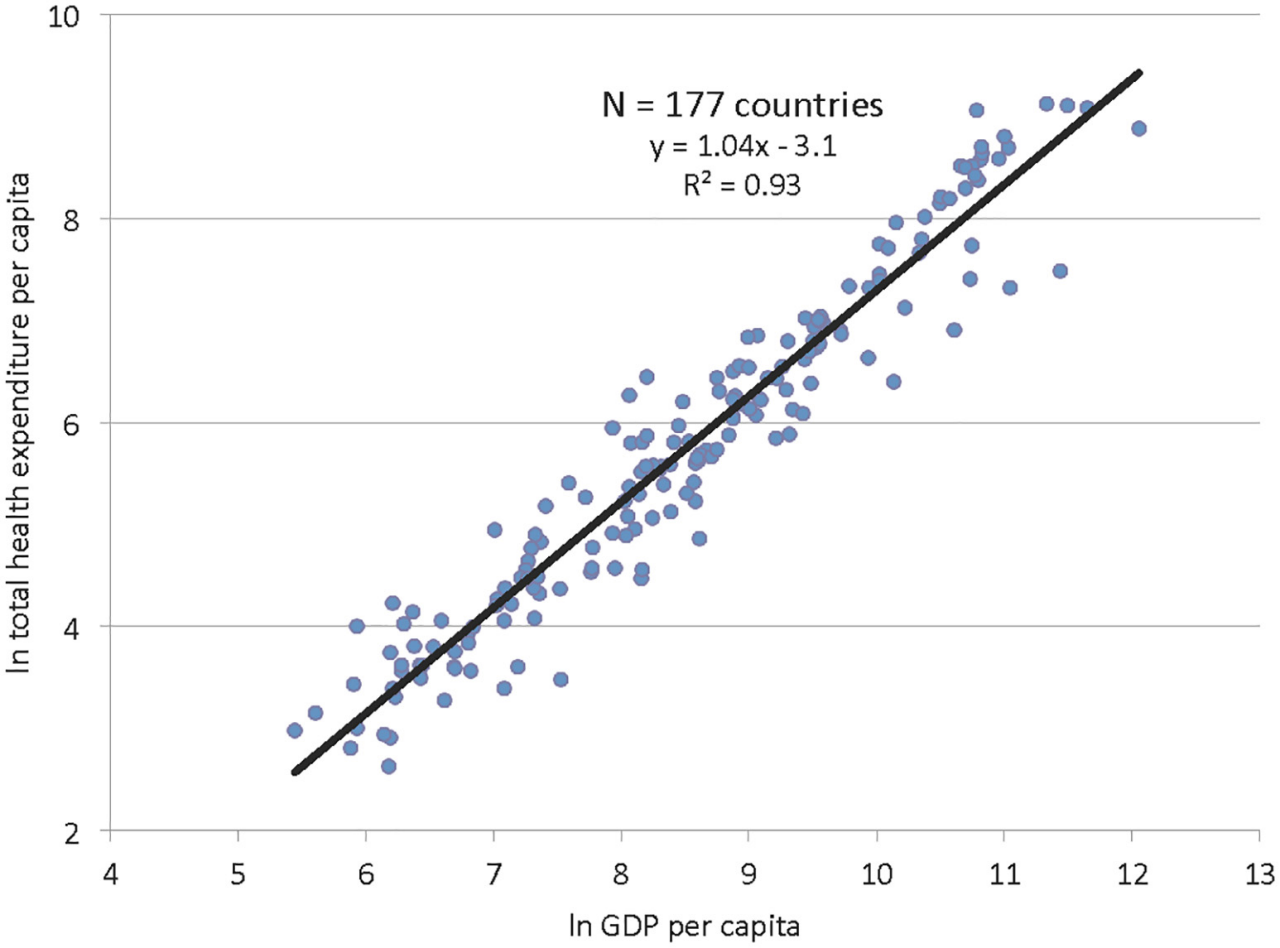
The relationship between country income and national health spending. Data are from http://data.worldbank.org/data-catalog/world-development-indicators. *Image credit*: *The Commission on Investing in Health*.

The report estimates the funding mix over time using the assumptions that (a) public health spending will steadily grow from present levels (2% of GDP in LICs and 1.7% in LMICs) to 3% of GDP by 2035, and (b) up to two-thirds of the increment in public spending on health could be allocated to the convergence agenda. The assumption that spending on health will rise as GDP rises is based on empirical research that shows an association between rising GDP and increasing public health spending—an association known as the “first law of health economics.” Fig 2 shows the results of an analysis by the CIH, presented in the CIH report [1], of the association between GDP and health expenditure across 177 countries.

We have used this same approach to estimate how the convergence agenda might be financed in the years leading up to 2035, assuming a linear increase in the share of GDP allocated to public spending on health over this time frame from a base year of 2011.

In LICs, the incremental cost of convergence is US$24 billion in 2015, increasing to US$27 billion in 2030 and US$30 billion in 2035. Given the need for very robust scale-up investments, the funding gap (i.e., the need for external finance) is high early on: US$22 billion in 2015. As LICs increase the share of their growing GDP to public spending on health, domestic financing is able to gradually cover more of the convergence. The financing gap falls from US$22 billion in 2015 to US$17 billion in 2020, US$12 billion in 2025, US$10 billion in 2030, and US$9 billion in 2035.

In LMICs, projected economic growth suggests significantly less need for external funding for health compared with LICs. In LMICs, the incremental cost of convergence is US$33 billion in 2015, increasing to US$53 billion in 2030 and US$61 billion in 2035. Our calculations estimate that there is a funding gap in 2015 of about US$2 billion, which donor financing could easily meet. After this, as public spending on health increases, the cost of convergence can be covered entirely by domestic financing.

Such a transformation in global health will, however, only be possible if the very large “delivery gap” can be closed—that is, the gap between the interventions known to be effective and what is actually being delivered [3]. Closing this gap, also termed the “know-do” gap [3] or the “coverage gap” [4], has been a particularly stubborn global health challenge. The coverage gap is exacerbated by a “technical capacity gap” in the highest-burden countries, in which national planning and implementing bodies (usually the ministry of health) are often understaffed and underfunded, resulting in limited capacity to define health priorities, appraise and use scientific evidence, and plan and evaluate health programs.

An example of the delivery gap is the treatment for childhood diarrhea, the world’s second leading cause of child mortality, responsible for about 600,000 deaths in 2013 [5]. Universal coverage with oral rehydration therapy (ORT), which costs just pennies, would reduce these deaths by over 90% [6], but only around four in 10 children receive this treatment. In zinc-deficient populations, zinc treatment reduces diarrhea-related child deaths by about a quarter [7], yet only 1% of affected children receive treatment [2]. Even though many countries have formally adopted diarrhea treatment policies, “there is a gap between policy change and effective programme implementation with very few children currently being appropriately treated” [8]. Overall, up to 85% of all maternal, neonatal, and child deaths in LICs and LMICs could be averted through scale-up of such existing health interventions [9].

In this essay, we examine policy and implementation research (PIR), the scientific enterprise aimed at closing this delivery gap. The term PIR captures both the emerging field of implementation research, or implementation science, and its sister domain, health policy and systems research. We discuss the value proposition of PIR for donor, low-, and middle-income countries. We propose the roles that (a) national governments, institutions, and researchers and (b) international institutions should play over the next 20 years to foster PIR. Lastly, we propose a PIR research agenda for the period 2015–2035. Tackling the key questions on this agenda would go a long way to achieving the vision of convergence and the other major global health goals laid out in *Global Health 2035*, such as curbing non-communicable diseases (NCDs) and injuries and achieving universal health coverage (UHC). Throughout this essay, we illustrate our arguments by giving case studies of how PIR has shaped global health practice and outcomes.

## Policy and Implementation Research: An Urgent Health Priority

When national and global health researchers are asked to prioritize the key research questions that must be addressed to reduce avertable mortality, questions related to implementation are usually scored the highest. For example, when child health experts conducted a research prioritization exercise with a focus on reducing childhood deaths from diarrhea worldwide, the highest ranked questions were those related to overcoming barriers to use of existing treatments, particularly ORT and zinc [10]. When technical experts ranked the most important research investments to reduce child mortality in South Africa, the top three priorities out of 63 options were PIR to scale up coverage with (1) vitamin A supplementation, (2) hand washing with soap, and (3) antibiotics for pneumonia [11]. In addition to researchers, stakeholders for PIR include communities that stand to benefit from effective interventions, as well as practitioners who deliver care and policymakers who allocate funds and are accountable for good health outcomes.

As Whitworth and colleagues have argued, “we do not know how best to scale up interventions effectively” [9], and we urgently need to improve our knowledge. PIR is the scientific research enterprise that aims to generate generalizable evidence on what works to implement effective programs and policies. A key area of focus for PIR is identifying which scale-up strategies are the most effective and which contextual, population, and policy factors are associated with scaling success [12–14]. It has two interrelated domains:

- Implementation research [12], the first domain, is defined as research that “aims to develop strategies for available or new health interventions in order to improve access to, and the use of, these interventions by the populations in need” [15]. This research is applied when interventions are already proven to work but it is unclear how best to effectively ensure their adoption and integration into a health system (Box 2) [16,17]. An important characteristic of implementation research is that it produces generalizable knowledge about effective delivery that can be applied to many health programs (in contrast to operations research, which aims to solve operational problems facing an individual, local program) [18,19].

**Box 2. Key Components of Implementation Research**

The Implementation Science Alliance, convened by the United States National Institutes of Health and the US President’s Emergency Plan for AIDS Relief (PEPFAR), focuses on reducing vertical transmission of HIV. The alliance sees implementation research as having four key components: “(1) understanding the implementation environment; (2) studying the actual process of implementation; (3) testing innovative implementation approaches; and (4) linking evidence from such research to policymaking, program implementation, and scale-up” [16].

The World Health Organization (WHO) Implementation Research Platform sees implementation research as “research that:

- Identifies common implementation problems and their main determinants that hinder effective access to interventions;
- Develops and tests practical solutions to tackle these problems that are either specific to particular health systems and environments or address a common problem faced by several countries in a region; and
- Determines the best way of introducing potential practical solutions into the health system and facilitates their full-scale implementation, evaluation and modification as required” [17].

- Health policy and systems research, the second domain, is defined by the Alliance for Health Policy and Systems Research (AHPSR) as a “field that seeks to understand and improve how societies organize themselves in achieving collective health goals, and how different actors interact in the policy and implementation processes to contribute to policy outcomes” [20]. This form of research examines the way in which scale-up is affected by broad systemic and policy factors, such as health financing, human resources, and the quality of care.

*Global Health 2035* made a powerful case for increasing investments into developing new health tools and also expanding the use of PIR to guide scale-up of new and existing tools. There is currently very little funding for PIR. The AHPSR estimates that only around 0.02% of health expenditure in low- and middle-income countries is devoted to this type of research [20]. This is a result of both insufficient overall research spending as well as the low priority for PIR. One study of child health research grants from the US National Institutes of Health and the Bill & Melinda Gates Foundation found that 97% of research funding is directed at developing new health technologies and just 3% at implementation research [21]. Commenting on what they call the “3/97 gap” [21], the authors conclude that “there is a serious discrepancy between current research and the research needed to save children's lives.” They estimate that research on developing new technologies could avert about 22% of child deaths, less than a third of the deaths that could be prevented through improvements in scaling up existing tools.

In other words, rapidly saving more lives will be achieved through a better understanding of what one of us has called the “determinants of performance,” i.e., the factors that lead to successful delivery of health interventions in a way that maximizes health outcomes [22]. These factors may include features of the intervention and the local health systems, organizational culture, and user preferences and needs. This knowledge will, in turn, facilitate policy- and decision-makers in achieving efficiency when it comes to investing in health, as it highlights which investments—or combinations of investments—will make the most difference in public health outcomes.

### The Value of PIR

For national actors, such as policymakers, health providers, and program managers, a key value proposition of PIR is that it provides solutions to overcoming implementation bottlenecks in their own national health systems [23]. National support to build capacity in PIR, including by creating a conducive research environment, enables individuals and institutions to tackle key health issues on the ground [9]. For international actors, including multilaterals, bilateral development organizations, and foundations, the value of PIR is in (a) learning about which implementation models work best for different settings, (b) helping to develop a menu of context-specific policy options from aggregating national experiments, and (c) allowing local policymakers to retain ownership and responsibility for improving outcomes and impacts. PIR can help to identify the leverage points needed to improve the ability of any given intervention or program to fully realize its potential, which, in turn, drives efficiencies by giving motivated decision makers evidence on “doing more with less” (i.e., saving more lives with less money).

Research generated locally can, in many cases, support global learning. This was seen in (a) Mexico’s large-scale health reform process (Box 3), which began in 2000 and which generated influential policy insights for other countries [23,24]; (b) the use of PIR from other settings in rebuilding Rwanda’s health system after the 1994 genocide (Box 4) [25]; and (c) the second phase of the US President’s Emergency Plan for AIDS Relief (PEPFAR II), which ran from 2009 to 2013 after an initial phase aimed at rapid delivery of HIV interventions [26]. In PEPFAR II, PIR was used to guide strategy, eliminating the use of prevention interventions that were low-impact (as measured by the incidence of new infections) and expanding treatment services from 1.7 million people to 7.8 million people, with a 15% drop in PEPFAR funding [26–29]. Global health research can also generate knowledge that can be brought into national health policymaking, while PIR on domestic reforms can, in turn, feed back into the “global pool of experience” [23].

**Box 3. Policy and Implementation Research in Action: The Mexican Health Reforms**

Beginning in 2000, Mexico embarked on an ambitious program to dramatically scale up (a) coverage with key health interventions to improve public health outcomes, and (b) publicly financed insurance coverage to reduce household poverty from out-of-pocket medical expenses. At every step along the way, the results of both international and national PIR informed the reform process [23].

The reforms were prompted by the unexpected findings of both national and international research. First, calculation of Mexico’s national health accounts showed that over half of the population was paying for health expenses out of pocket, and these expenses were regressive, representing a higher percentage of income of the poor than the rich. Second, an international study that compared health systems performance across countries found that Mexico did very poorly on measures of “fair financing” (a metric that assesses whether people are contributing more as their income level rises). This international study, in turn, led Mexico’s ministry of health to conduct additional research that showed that poor, uninsured households were the most likely to have suffered catastrophic medical expenses.

This “careful interplay between national and international analyses” [23] provided data to support the 2004 rollout of a new publicly financed health insurance scheme called *Seguro Popular* (Popular Health Insurance). The scheme funds a package of health interventions that were chosen based on evidence on cost, effectiveness, and acceptability, and that particularly benefitted women and children. In the ten years since its launch, 51 million people have been enrolled in the scheme, meaning that Mexico has achieved universal health coverage (UHC).

*Seguro Popular* built on a previous Mexican initiative aimed at scaling up health and education interventions called *Progresa* (Progress), later renamed as *Oportunidades* (Opportunities). The initiative was launched in the mid-1990s and was itself informed by national PIR. It offers cash transfers to mothers on the condition that they attend a clinic to receive maternal and child health care (e.g., vaccinations, family planning, and nutritional support) and that their children go to school. A “rigorous and politically neutral evaluation of the program” [24] showed several benefits, such as reduced childhood stunting, as well as areas for improvement. The evaluation results, in turn, fed back into the program over time.

**Box 4. How Rwanda Is Using PIR to Rebuild Its Health System**

The 1994 genocide in Rwanda caused a collapse in the country’s health system, leading to the world’s highest child mortality rate and lowest life expectancy. Over the last 20 years, the transformation of the health sector, guided by a policy document called *Vision 2020*, has been extraordinary. The rebuilding has been shaped by PIR at multiple points along the way. Rwanda’s minister of health, Agnes Binagwaho, and colleagues recently described the country’s two main PIR approaches [25].

The authors call the first PIR approach “disciplined experiments” to learn from, improve, and scale innovations in care delivery. Examples include:

- Piloting and evaluating performance-based financing (PBF) for HIV care delivery and maternal and child health services before being taken to national scale (PBF involves payments for achieving particular results, such as increased vaccination rates or facility-based births, rather than just paying for inputs, such as equipment and staff time);
- Piloting and evaluating community-based health insurance before being rolled out nationwide;
- Comprehensive health systems strengthening, including quality improvement and integrated platforms of community-based district health services, which is being evaluated using population-level outcomes data; and
- Engaging community health workers in HIV care (e.g., conducting daily visits and providing nutritional support), a strategy called “accompaniment;” this approach is being evaluated through cohort studies.

The second PIR approach is rigorous evaluation of health systems innovations, such as task-shifting of HIV care from physicians to nurses and community health workers, school-based HPV vaccination, providing food and transport to patients with multi-drug-resistant tuberculosis (TB), and quality improvement monitoring of national malaria and integrated TB–HIV programs.

All too often, governments and donors have funded the rollout of a national health program or significant health system reforms in a country without conducting a rigorous evaluation, which is not only a missed opportunity to improve local outcomes but a wasted opportunity for other countries to learn. As a starting point, funders and researchers should build a high-quality global database of PIR results, presented in a format sensitive to end users’ needs; as a result, donors and countries themselves will be less likely to waste scarce resources on recycling failed approaches and will be more likely to have data at their fingertips to improve decisions (Box 5) [30]. To make use of such data, ministries of health need to build national capacity to interpret and manage PIR data for monitoring and evaluation and, conversely, directly engage in shaping the PIR research agenda. This, in turn, would create a positive feedback loop in the medical delivery system. The ministry would become more responsible in the use of the resources under its control and would be able to modify programs based on the PIR findings, ensuring that any new resources are truly additive over the duration of a program.

**Box 5. Recycling Failed Approaches: Brazil’s Experience with Flour Fortification to Tackle Child Anemia**

Over the past two decades, prevalence studies have suggested that up to 80% of Brazilian children under the age of 5 years have anemia, and half of these cases are likely to be due to dietary iron deficiency. In an attempt to tackle iron-deficient anemia, in July 2004, the government instituted a national mandate that wheat and corn flours should be fortified with iron. The mandate specified that 4.2 mg of iron should be added to every 100 g of flour, corresponding to about half the daily recommended amount of iron for children aged under 5 years [30].

However, the government did not first pilot the approach before taking it to national scale (Cesar Victora, personal communication). A series of independent evaluations of the national scale-up program, involving four population-based surveys conducted from 2004 to 2008, found no impact of fortification on anemia, and an increase in anemia prevalence among children 2 years of age. The lack of impact was probably due to manufacturers using low-bioavailability reduced iron and adopting other poor fortification practices, together with a high prevalence of non-iron deficiency anemia. The authors conclude that “the national programme was ineffective as implemented” [30].

This was not the first failed national program of this kind. A cross-country analysis of national programs of iron fortification of wheat flour across 78 countries concluded that only nine programs were likely to have a positive impact if coverage could be optimized. As in Brazil, most countries were using non-recommended, low-bioavailability forms of iron.

PIR can be valuable in protecting governments and donors from “recycling failure.” Iron fortification programs will continue to fail unless policies are changed: fortification will only work if adequate amounts of high-bioavailability iron are used.

As one of us has previously argued, at its best, PIR involves “systematic and rigorous analysis of which delivery approaches worked across a variety of health needs and which did not” [18]. Such data can form the basis for developing successful implementation models that can be tested. PIR can also enhance efficiency by looking across multiple diseases and leveraging limited research funds to find ways to improve health system delivery of integrated packages of interventions and services. In addition, by guiding domestic and donor funding toward scaling up programs that have previously been shown to work, it also improves accountability for investments that are being made with the aim of improving people’s health. As shown in Table 2, a greater focus on PIR will help national and international actors to better respond to the challenges that are accompanying the recent shifts in the global health landscape and to anticipate future shifts (particularly a shift in which people are more likely to suffer from chronic, noncommunicable, and disabling conditions than from acute fatal infections) as well as shifts in the political, cultural, and economic contexts in LMICs. Finally, PIR is an essential tool for accountability to the population for resources spent and outcomes delivered.

**Table 2:** Ways in which PIR can help respond to the challenges associated with the shifts in the global health landscape.

Shift in the landscape	Challenge posed by this shift	How PIR can help respond to the challenge
Rapid economic growth of LICs and MICs and associated rise in domestic spending on health	Ensuring that the new funds for health are used effectively and efficiently to expand coverage of key interventions	Results from PIR can guide ministries of health and financing in supporting effective approaches to scale-up
“Dual burden” of infectious and non-communicable diseases in LICs and MICs	Scaling up health tools and services for a broad array of different conditions that have a wide variety of risk factors	PIR produces generalizable knowledge on effective delivery of interventions for both infections and NCDs (e.g., knowledge on how to scale up HIV services that can be applied to scaling up diabetes and hypertension care)
Recent national health reforms in LICs and MICs aimed at expanding health insurance	Financing and delivering insurance through approaches that are efficient and equitable and that reduce impoverishment from medical expenses	PIR can help guide the implementation of financing of insurance reforms, including the design of the benefits package and the best ways to protect the poor
Growing realization among donors that they need to support health systems strengthening, especially in LICs	Transitioning donor support away from funding unique, parallel systems (“vertical programs”) that finance and deliver single health interventions (e.g., antiretroviral drugs) toward funding comprehensive health systems	PIR can generate knowledge on how best to use development assistance for health to support health systems (e.g., guiding the transition of a donor’s support for HIV programs toward broader system strengthening)

## How to Boost PIR Capacity—Nationally and Globally

In the previous sections, we made the case that (a) underinvestment in PIR is costing lives and potentially wasting resources on inefficient models, and (b) greater attention and funding for PIR would help meet many of the complex health challenges facing governments and the broader global health community. But what would it take to enhance the capacity to conduct rigorous PIR?

Below, we briefly consider the respective roles and responsibilities of (1) national governments, institutions, and researchers, and (2) international institutions and researchers.

### National Action to Support PIR

The capacity to conduct national PIR requires a solid substrate of commitment, knowledge, and infrastructure. Governments, especially the ministries of health and finance, must evolve their culture to incorporate the use of PIR in their budgetary and strategic planning. Governments must foster the right research environment, which includes dedicated government funding for PIR (e.g., Brazil and Mexico are two countries that have made commitments to support PIR). Commitment to research and demand for data at the highest levels of government can be instrumental in instilling and supporting a culture of evidence in line ministries. The 2008 Bamako Call for Action, arising from the Global Ministerial Forum for Health Research in Mali, committed governments to spending at least 2% of their health ministry budgets on all health research; building research infrastructure and fostering the next generation of young health researchers were specifically included in the call to action [31]. Few countries have met their commitments. Going through the exercise of PIR-informed planning and budget development will support a culture of accountability in achieving certain outcomes for specific populations that will enhance decision-makers’ ability to understand who is benefiting (or not) from the interventions.

Linking the investment with the specific outcome to generate a specific impact is the appropriate level of specificity for the ministry of health (i.e., national, provincial or state, district, and village levels).

Governments should integrate high-quality evaluation (not just monitoring) into the rollout of programs, policies, and service innovation; this will permit course correction and will generate generalizable knowledge. Governments should not use or “capture” PIR to help support their own political agendas. Instead, the research they fund must be independent enough to permit negative findings and rigorous enough to provide generalizable lessons. They should foster a research environment in which individual and institutional conflicts of interest are carefully considered, disclosed, and addressed (especially in the situation in which an implementing agency wishes to evaluate its own work, given that such evaluations are prone to bias [18]). Transparency should be a key goal, including sharing data and results, in order to maximize public accountability and audit, regardless of where the research is conducted (i.e., by government, academic, or private research entities).

The researchers conducting PIR should, of course, interact with policymakers to identify key research priorities and to ensure that the research informs national planning and implementation processes. For maximum policy impact, health researchers should align their studies with national health priorities.

All research institutions should also make PIR results publicly available through a readily accessible repository. These data should be seen as a public good that can (a) guide future research, (b) promote government accountability to citizens, health providers, and health researchers, and (c) contribute to national and global knowledge banks.

As Whitworth and colleagues have argued, there is a need to “broaden the base” for health research in low- and middle-income countries, especially for PIR [9]. They argue that a first step toward building PIR capacity is to “listen to the voices of those grappling with the issues on the ground.” At the national level, these voices have become increasingly organized into a number of research initiatives, think tanks, or centers of excellence, many of which are now collaborating through regional networks. Governments should support these centers of PIR innovation. For example, 2007 saw the launch of the Initiative to Strengthen Health Research Capacity in Africa, which aims to create “self-sustaining pools of excellence” to conduct high-quality health research in Africa that can lead to systemic change [32]. In 2008, an alliance of seven schools of public health in central and east Africa was formed, called the Africa Hub, to build and strengthen PIR capacity [33].

### International Action to Support PIR

The international community has placed very little priority on PIR to date. The *Global Health 2035* report called for much greater funding for this kind of research—indeed, international research funders must help to redress the “3/97” gap. The report argued that the capacity to conduct PIR “can be strengthened—and the results more quickly disseminated—by a well-funded mix of South–South and South–North collaborations” [1]. It also urged donors to support national capacity to conduct PIR in LICs and LMICs as well as international collaborative PIR networks. Investing in local research organizations is important both for expanding the reach of PIR and for promoting local uptake of findings [34]. The international community can also play an important normative role in encouraging domestic investments in health research.

Global health investors should lead by example by insisting that their initiatives undergo rigorous independent evaluation and by publicly sharing the evaluation results. As *The Lancet* editors argued, the “massive scale-up in global health investments during the past decade has not been matched by an equal commitment to evaluation” [35].

### A PIR Research Agenda for 2015–2035

In the final section of this essay, we propose an initial PIR research agenda for supporting the goal of a grand convergence by 2035 and the other major goals of the *Global Health 2035* report. This agenda reflects the collective ideas of the authors, who have all been involved in global health implementation activities and who participated in a six-month consultative process to define priority PIR questions. We acknowledge that the agenda is not comprehensive—our aim was to put these ideas on the table to stimulate further discussion and refinement. We have grouped our suggested priorities into five categories: (1) closing the delivery gap to achieve convergence and to control NCDs and injuries, (2) population-based approaches to reduce the risks of NCDs and injuries, (3) reaching UHC, (4) ensuring that the poor benefit, and (5) building the foundation for evidence-based policy.

### Closing the Delivery Gap to Achieve Convergence on Infectious Diseases and Maternal/Child Health and Control NCDs and Injuries

- Identify (a) under-delivered interventions (e.g., low rates of coverage with ORT or zinc for diarrhea, skilled birth attendance, or cervical cancer screening); (b) weak points in service delivery, including operational and policy bottlenecks; and (c) causes of low coverage (e.g., issues of access, retention of patients in programs, and poor-quality services).
- Identify ways to overcome these delivery bottlenecks by improved delivery strategies; such research could include examination of the potential role of (a) integrating services (e.g., HIV with hypertension and/or diabetes care); (b) human resource needs, including task shifting and provider extension strategies; (c) community mobilization and engagement (e.g., delivery of services by community health workers linked to a formal referral and delivery system); (d) management of cohorts of patient populations with similar conditions; and (d) improved access to and supply of high-quality, low-cost medicines, vaccines, fit-for-purpose technology, and other health tools.
- Estimate the costs, cost-effectiveness, and cost–benefit ratio of different delivery strategies to achieve convergence.
- Test strategies for improving the performance of health workers in delivering high-quality services (e.g., accreditation of providers, financial incentives, supervision programs, improved leadership, team-based care).
- Identify the technical assistance needs that would effectively expand the capacity of the country and its ability to manage, oversee, monitor, and evaluate programmatic impact. It is critical to understand the way in which donor, multilateral, and foundation support contributes to sustaining and expanding effective and beneficial programming. Such funding clearly needs to be secure.

### Population-Based Approaches to Reduce the Risks of NCDs and Injuries

- Review existing data on taxation of tobacco, alcohol, and sugar to identify (a) the most effective policies, (b) the lessons from one country that could be applied to other countries, and (c) the lessons from tobacco taxation that could be applied to taxing other harmful substances (e.g., sugar-sweetened beverages, highly processed foods).
- Define the elements of a comprehensive tobacco control program (national, country-specific PIR can show the role of national contextual factors).
- Define the essential clinical and public health interventions that maintain and advance health in the population, e.g., immunizations, nutrition counseling (including defining an acceptable diet in cultures that are dominated by starch-based foods), reduction of preterm deliveries, effective family planning, injury prevention strategies, cancer screening, screening for and management of hypertension and diabetes, and screening for coronary artery disease.
- Identify how population-based policies targeting key risk factors can be effectively scaled (e.g., promoting exercise, calorie labeling, reducing the content of salt, or eliminating trans fatty acids in foods) to prevent NCDs, and determine which approaches are most appropriate for specific contexts.

### Reaching UHC

- Define a standard benefit package in each country using best epidemiologic data and projections and incorporating population expectations and preferences, and cost this package so that the incremental funding required can be defined. This costing will be essential for mobilizing domestic and donor resources for UHC.
- Evaluate country experience of insurance reforms to expand coverage and financial protection to assess impacts and learn implementation lessons (the World Bank recently published a good example of a systematic review on the impact of UHC schemes [36]).
- Examine priority policy questions for UHC, such as which types of insurance models and benefit packages are most effective in expanding coverage and improving health outcomes and which are the best ways to scale insurance in heterogeneous settings (e.g., federal systems, decentralized systems).
- Evaluate health systems reforms to improve efficiency of care delivery to ensure equity and control costs as demand for health rises (Berman and Bitran have argued that evaluating such reforms should be “an integral part of good practice in health system strengthening efforts to guide planning, policy development, monitoring, and evaluation” [37]). Examine the role of strategic purchasing (purchasing of services based on population health needs and provider quality and efficiency [38]) in improving health outcomes, including purchasing from private providers.
- Identify mechanisms for assuring broad scale interoperability of information systems to allow for the rapid exchange of patient information to improve patient quality of care, and support ongoing PIR related to health systems change.
- Establish best practices for engaging the non-state sector (private providers, nongovernmental organizations, etc.) in expanding service coverage and quality, including the role of social franchising (in which private providers are organized into “networks that deliver specified health services under a common brand, with a promise of quality assurance” [39]).

### Ensuring That the Poor Benefit

- Identify the most effective and efficient ways to identify and reach the poor and others experiencing health disparities with health services and insurance; assess whether existing programs are pro-poor and equitable.
- Understand the range and magnitude of financial barriers for the poor and near-poor, from direct medical costs such as clinic fees and medicines to ancillary costs of transport and lodging; assess policy options for reducing these costs while maintaining adequate health facility financing.
- Test whether financial interventions, such as conditional cash transfers (Box 3) and performance-based funding (Box 4) [40], can improve health outcomes among disadvantaged groups, and whether integrating these interventions into primary care services is effective [41].

### Building the Foundation for Evidence-Based Policy

- Identify the health policymaking processes and drivers in each country—identify who defines the populations in need, who sets health investment priorities, and how best donor resources can be incorporated into national policymaking without distorting national priorities.
- Evaluate the effectiveness of national centers of excellence and regional networks that focus on PIR and health systems strengthening.
- Evaluate the role of scorecards and other standardized tools and models in encouraging governments to improve public health and health care delivery (scorecards compare how different countries are performing against each other in scaling up particular health services [42,43]).

## Conclusion

The global health landscape is undergoing an important shift. Over the last 15 years, the health community has shown that complicated and sometimes progressive diseases, including HIV/AIDS and TB, can be prevented, diagnosed, and treated through targeted funding of high-impact interventions, matched with the technical knowledge to implement them in a cost-effective manner. *Global Health 2035* showed that, collectively, governments worldwide have the financial and technical capacity to achieve dramatic gains in health within a generation. This transformation will be aided by the global move toward UHC, fueled by rapid economic growth and rising population aspirations for better health in rich and poor countries alike. Delivering on this aspiration, however, will require a concerted and intense program of national and international PIR. We hope that our suggested research agenda will help to stimulate such crucial research.

This collection of translational science essays has focused mostly on the new health technologies that will be needed to achieve a grand convergence in global health. The billions of dollars in investments in discovering and developing new tools must be accompanied by a similar effort to improve delivery and implementation. PIR to address “the challenges of effective and sustainable implementation of proven interventions in real-world settings” [15] will be critical for global health transformation.

## Abbreviations

AHPSR: Alliance for Health Policy and Systems Research
CIH: Commission on Investing in Health
LIC: low-income country
LMIC: lower-middle-income country
NCD: non-communicable diseases
ORT: oral rehydration therapy
PBF: performance-based financing
PEPFAR: President’s Emergency Plan for AIDS Relief
PIR: policy and implementation research
TB: tuberculosis
UHC: universal health coverage
WHO: World Health Organization

## References

[1] Jamison DT, Summers LH, Alleyne G, Arrow KJ, Berkley S, et al Global health 2035: a world converging within a generation. *Lancet* 2013; 382:1898–955. 10.1016/S0140-6736(13)62105-4 24309475

[2] Stenberg K, Axelson H, Sheehan P, et al Advancing social and economic development by investing in women’s and children’s health: a new Global Investment Framework. *Lancet* 2014; 383:1333–54. 10.1016/S0140-6736(13)62231-X 24263249

[3] WHO (2006). Bridging the “Know–Do” Gap. Meeting on Knowledge Translation in Global Health, 10–12 October 2005, Geneva, Switzerland. http://www.who.int/kms/WHO_EIP_KMS_2006_2.pdf

[4] Friberg IK, Kinney MV, Lawn JE, Kerber KJ, Odubanjo MO, et al Sub-Saharan Africa's mothers, newborns, and children: how many lives could be saved with targeted health interventions? PLoS Med 2010;7(6): e1000295 10.1371/journal.pmed.1000295 20574515 PMC2888572

[5] Liu L, Oza S, Hogan D, Perin J, Rudan I, et al Global, regional, and national causes of child mortality in 2000–13, with projections to inform post-2015 priorities: an updated systematic analysis. *Lancet*. 2015;385:430–440. 10.1016/S0140-6736(14)61698-6 25280870

[6] Munos MK, Fischer Walker CL, Black RE. The effect of oral rehydration and recommended home fluids on diarrhoea mortality. Int J Epidemiol 2010;39(suppl 1):i75–87. 10.1093/ije/dyq025 20348131 PMC2845864

[7] Fischer Walker CL, Black RE. Zinc for the treatment of diarrhoea: effect on diarrhoea morbidity, mortality, and incidence of future episodes. Int J Epidemiol 2010;39(suppl 1):i63–9. 10.1093/ije/dyq023 20348128 PMC2845862

[8] Fischer Walker CL, Fontaine O, Young MW, Black RE. Zinc and low osmolarity oral rehydration salts for diarrhoea: a renewed call to action. *Bull World Health Organ* 2009;87(10):780–6. 19876545 10.2471/BLT.08.058990 PMC2755312

[9] Whitworth J, Sewankambo NS, Snewin VA. Improving implementation: building research capacity in maternal, neonatal, and child health in Africa. *PLoS Med* 2010;7(7): e1000299 10.1371/journal.pmed.1000299 20625547 PMC2897765

[10] Fontaine O, Kosek M, Bhatnagar S, Boschi-Pinto C, Chan KY, et al Setting research priorities to reduce global mortality from childhood diarrhoea by 2015. *PLoS Med* 2009;6(3): e1000041. 10.1371/journal.pmed.1000041 PMC2653551 19278292

[11] Tomlinson M, Chopra M, Sanders D, Bradshaw D, Hendricks M, et al Setting priorities in child health research investments for South Africa. *PLoS Med* 2007;4(8): e259 17760497 10.1371/journal.pmed.0040259 PMC1952202

[12] Madon T, Hofman KJ, Kupfer L, Glass RI. Public health. Implementation science. *Science* 2007;318:1728–1729. 18079386 10.1126/science.1150009

[13] Victora CG, Schellenberg JA, Huicho L, Amaril J, El Arifeen S, et al Context matters: interpreting impact findings in child survival evaluations. Health Policy Plan 2005 12;20 Suppl 1:i18–i31. 16306066 10.1093/heapol/czi050

[14] Svoronos T, Mate KS. Evakuating large-scale health programmes at a district level in resource-limited countries. *Bull*. *World Health Org* 2011;89:831–37 22084529 10.2471/BLT.11.088138 PMC3209726

[15] Remme JHF, Adam T, Becerra-Posada F, D'Arcangues C, Devlin M, et al Defining research to improve health systems. *PLoS Med* 2010;7(11): e1001000 10.1371/journal.pmed.1001000 21124816 PMC2993153

[16] Sturke R, Harmston C, Simonds RJ, Mofenson LM, Siberry GK, et al A multi-disciplinary approach to implementation science: the NIH-PEPFAR PMTCT Implementation Science Alliance. *J Acquir Immune Defic Syndr* 2014; 67 (suppl 2):S163–S167 10.1097/QAI.0000000000000323 25310124

[17] World Health Organization. Implementation Research Platform: Call for Proposals for Implementation Research. http://www.who.int/alliance-hpsr/alliancehpsr_irpcallresearch.pdf?ua=1

[18] Kruk ME. More health for the money—toward a more rigorous implementation science. *Sci*. *Transl*. *Med*. 2014;6 (245): 245ed17 10.1126/scitranslmed.3009527 25031266

[19] World Health Organization. Alliance for Health Policy and Systems Research: About Us. http://www.who.int/alliance-hpsr/about/en/

[20] Alliance for Health Policy and Systems Research (2004) Strengthening health systems in developing countries: The promise of research on policy and systems. Geneva: Alliance for Health Policy and Systems Research http://www.who.int/alliance-hpsr/resources/Strengthening_complet.pdf

[21] Lero JL, Habicht JP, Pelto G, Bertozzi SM. Current priorities in health research funding and lack of impact on the number of child deaths per year. *Am J Public Health* 2007;97:219–23. 17194855 10.2105/AJPH.2005.083287 PMC1781402

[22] Goosby EP. Global Health Delivery and Diplomacy: The Long Road to Sustainable Programs. http://cfar.ucsd.edu/links/downloads/seminar-slides/goosby-chris-20141001

[23] Frenk J. Bridging the divide: global lessons from evidence-based health policy in Mexico. *Lancet* 2006; 368: 954–961. 16962886 10.1016/S0140-6736(06)69376-8

[24] Skoufias E. PROGRESA and Its Impacts on the Welfare of Rural Households in Mexico. Washington, DC: International Food Policy Research Institute 2005 http://ageconsearch.umn.edu/bitstream/37891/2/rr139.pdf

[25] Binagwaho A, Farmer PE, Nsanzimana S, Karema C, Gasana M, et al Rwanda 20 years on: investing in life. *Lancet* 2014; 384: 371–75. 10.1016/S0140-6736(14)60574-2 24703831 PMC4151975

[26] Pepfar Blueprint: Creating an AIDS-Free Generation. November 2010. http://www.pepfar.gov/documents/organization/201386.pdf

[27] Padian NS, Holmes CB, McCoy SI, Lyerla R, Bouey PD, Goosby EP. Implementation science for the US President's Emergency Plan for AIDS Relief (PEPFAR). J Acquir Immune Defic Syndr 2011;56:199–203. 10.1097/QAI.0b013e31820bb448 21239991

[28] US Department of State. PEPFAR announces implementation science awards. August 1, 2012. http://www.state.gov/r/pa/prs/ps/2012/08/195936.htm

[29] Holmes CB, Blandford JM, Sangrujee N, Stewart SR, DuBois A, Smith TR, Martin JC, Gavaghan A, Ryan CA, Goosby EP. PEPFAR's past and future efforts to cut costs, improve efficiency, and increase the impact of global HIV Programs. Health Affairs 2012;31:1553–60 10.1377/hlthaff.2012.0562 22778345

[30] Assunção MC, Santos IS, Barros AJ, Gigante DP, Victora CG. Flour fortification with iron has no impact on anaemia in urban Brazilian children. Public Health Nutrition 2012;15(10), 1796–1801 10.1017/S1368980012003047 22704130

[31] The Lancet (editorial). The Bamako call to action: research for health. *Lancet* 2008; 372:1855 10.1016/S0140-6736(08)61789-4 19041784

[32] ISHReCA brochure (2008) http://www.mrc.ac.za/researchdevelopment/ISHReCAbrochure.pdf

[33] A collaborative endeavour: Strengthening institutional health systems research capacity for 7 Schools of Public Health in East and Central Africa. June 2, 2014. Health Research Policy and Systems. http://www.health-policy-systems.com/series/AfricaHub

[34] Holmes CB, Sikazwe I, Raelly R, et al Managing multiple funding streams and agendas to achieve local and global health and research objectives: lessons from the field. *J Acquir Immune Defic Syndr* 2014;65:S32–5. 10.1097/QAI.0000000000000043 24321983 PMC4122281

[35] The Lancet (editorial). Evaluation: the top priority for global health. *Lancet* 2010;375: 526 10.1016/S0140-6736(10)60056-6 20079530

[36] Giedion, Ursula; Alfonso, Eduardo Andres; Diaz, Yadira. 2013. The impact of universal coverage schemes in the developing world: a review of the existing evidence. Universal Health Coverage (UNICO) studies series; no. 25. Washington D.C.: The Worldbank.

[37] Berman P, Bitran R. Health systems analysis for better health systems strengthening World Bank HNP Discussion Paper. Washington, DC: The World Bank, 2011.

[38] Busse R, Figueras J, Robinson R, Jakubowski E. Strategic purchasing to improve health system performance: key issues and international trends. HealthcarePapers 8(Sp) September 2007: 62–76 10.12927/hcpap.2007.19221 http://www.longwoods.com/content/19221 19096267

[39] Social Franchising for Health. http://www.sf4health.org/

[40] Montagu D, Yamey G. Pay-for-performance and the Millennium Development Goals. *Lancet* 2011; 377:1383–5. 10.1016/S0140-6736(11)60544-8 21515147

[41] Guanais FC. The combined effects of the expansion of primary health care and conditional cash transfers on infant mortality in Brazil, 1998–2010. *Am J Public Health* 2013; 103: 2000–2006. 10.2105/AJPH.2013.301452 PMC3828713 24028257

[42] Rosinski AA, Narine S, Yamey G. Developing a scorecard to assess global progress in scaling up diarrhea control tools: a qualitative study of academic leaders and implementers. *PLoS ONE* 2013; 8(7): e67320 10.1371/journal.pone.0067320 23874412 PMC3706531

[43] The African Leaders Malaria Alliance (2011) ALMA Scorecard For Accountability and Action. http://www.alma2015.org/alma-scorecard-accountability-and-action.

